# Evaluating the reliability and validity of secondary reporting to measure gender-based violence in conflict and disaster

**DOI:** 10.1186/s13031-020-00301-0

**Published:** 2020-08-06

**Authors:** Lindsay Stark, Les Roberts, Gary Yu, Timothy M. Tan, Aishwarya Nagar, Alastair Ager

**Affiliations:** 1 Brown School at Washington University in St Louis, 1 Brookings Drive, St Louis, MO 63130 USA; 2 Mailman School of Public Health, 60 Haven Avenue, New York, NY 10032 USA; 3 NYU Rory Meyers College of Nursing, 433 First Avenue, New York, NY 10010 USA; 4 Department of Emergency Medicine, New York Health + Hospitals/Queens, 82-68 164th Street, Queens, NY 11432 USA; 5 Iris Group, Inc, 121 S Estes Drive, Suite 103C, Chapel Hill, NC 27514 USA; 6 Institute for Global Health & Development, Queen Margaret University, Edinburgh, EH21 6UU UK

**Keywords:** Gender-based violence, Intimate partner violence, Rape, Secondary reporting, Survey methods, Neighborhood method, Humanitarian, Conflict

## Abstract

**Background:**

Accurately identifying the magnitude of gender-based violence (GBV) in humanitarian settings is hindered by logistical and methodological complexities. The ‘Neighborhood Method’, an adapted household survey that uses primary and secondary reporting to assess the prevalence of GBV in humanitarian settings, reduces the length of time and cost associated with traditional surveys. Primary female adult respondents disclose incidents of physical violence, intimate and non-intimate partner rape for themselves, other females in their homes (standard reporting) and other women and children in their social networks (secondary reporting). This study examines the reliability and validity of this inclusion of secondary reporting to determine the comparability of the Neighborhood Method to a traditional survey approach.

**Methods:**

Drawing on data from 1180 women reporting on 3744 females in respondent households and 15,086 in neighboring households across four humanitarian settings (Ethiopia/ Somalia, Liberia, Sri Lanka, and Uganda), reliability of secondary reporting was measured through intra-class correlation coefficients (ICCs) and Cohen’s kappas. Validity was assessed using two-sample z-tests for differences between standard versus secondary reporting.

**Results:**

Prevalence estimates comparing a respondent’s household with a neighboring household show closer agreement (ICC: 0.999–0.986) than self-reports vs. secondary reporting on a female counterpoint in a neighboring home (ICC: 0.939–0.98). Kappa statistics analyzing the reliability of two separate neighbors reporting on a third neighbor showed moderate agreement beyond chance alone (κ = 0.45 for physical violence and 0.48 for rape). Prevalence rates corresponded between standard and secondary reports (i.e. showed no statistical difference) in 18 out of 24 compared populations.

**Conclusions:**

For prevalence of GBV, secondary reporting about neighbors can serve as a useful adjunct to standard survey methodology. Findings offer important initial insights into the consistency and accuracy of secondary reporting as a tool for field epidemiologists in humanitarian settings.

## Background

Accurate measurement of mortality, violence, and human rights violations in conflict- and disaster-affected populations is critical for informing advocacy, program response, resource allocation, and policy. This is especially true for gender-based violence (GBV), which is known to be prevalent in humanitarian emergencies and is detrimental to the health and wellbeing of vulnerable communities. GBV comprises acts (such as physical, emotional, psychological, or sexual violence) that are perpetrated against a person’s will and are based on unequal distribution of power, particularly gender inequities and norms [1]. GBV encompasses many types of violence, including sexual assault and coercion, physical violence, and intimate partner violence (IPV).

Traditionally, the humanitarian community has relied on qualitative and numerator-based service delivery data to inform programming and policy decisions related to GBV. However, such an approach does not provide a full picture of the scope and magnitude of GBV [2]. There are myriad complexities in collecting high quality population-based data on GBV in humanitarian settings, including ongoing instability, poor access to affected populations, and limited services to support survivors [3–5]. In addition to these logistical complexities, there are also numerous methodological challenges to measuring GBV accurately in such settings, such as underreporting due to fear or stigma, inconsistent operationalization of key outcomes, telescoping and issues with recall of past incidents [6–10].

In order to capture GBV data that are as reliable and accurate as possible in disaster-affected populations, we must first consider best practices in measuring this sensitive topic more generally. There is evidence, for example, that data quality is improved by making GBV the exclusive focus of a survey as opposed to embedding questions into broader surveys about reproductive or mental health [11]. Similarly, we know that survey instruments need to be tested and adapted to safely and adequately elicit incidents of GBV in different contexts. Additionally, survey instruments can address barriers like recall and telescoping by using a shorter recall period and identifying important local or national landmark events to help respondents identify when an incident took place [8, 12]. Matching interviewers based on gender and ethnicity has been shown to foster greater trust and rapport between participants and interviewers [12], and allowing for a longer interview schedule can similarly help to build trust and rapport [13]. Importantly, using a conversational, supportive, and non-judgmental style of interviewing can promote participants’ comfort disclosing sensitive information in a way in which they feel supported. For example, slow non-judgmental interviews with male and female couples produced highly consistent reporting of domestic violence among refugees in Jordan [14]. Self-reporting with a tablet or similar electronic device appears to have worked well in some settings [15]. Finally, and most critically, survey teams must consider and work to ensure participants’ safety at every stage of data collection [16].

While there is a reasonably developed body of evidence on good practice around fostering safe and valid disclosure of GBV in survey research, less well documented in the GBV literature are good practices related to sampling approaches. Acknowledging that numerator-based approaches are limited in their ability to ‘tell the whole story’, researchers often resort to the option of an expensive, time-intensive and logistically complex population-based sampling approach. Research into alternative sampling methods better suited to conflict and disaster settings are only beginning to emerge.

One promising approach is secondary reporting (sometimes called indirect sampling or proxy sampling), in which information is systematically gathered about ‘clusters’ of individuals from a respondent who hypothetically knows about the experiences of these other individuals [17]. Secondary reporting offers several potential advantages, including faster and more cost-effective data collection, increased sample size through a single interview, the opportunity to spend more time per interview with a respondent, which in turn reduces non-disclosure bias, and the ethical advantage of limiting the number of interviewees potentially exposed to further trauma or violence triggered by an interview [16]. At the same time, secondary reporting relies on a critical assumption: that informants can and will provide complete and accurate information about the experiences of others [10].

Our own previously published work on GBV employed secondary reporting in internally displaced persons (IDP) camps in Uganda [17], conflict-affected communities in Liberia [2], Somali refugee camps in Ethiopia [18], and conflict and tsunami-affected populations in Sri Lanka [19], but with limited attention to the reliability and validity of the data in comparison to standard self-report. In this article, we explore women’s knowledge and disclosure patterns about experiences of violence and examine whether secondary reporting can assess the magnitude of GBV in a valid and reliable way in conflict and disaster-affected settings.

## Methods

### Participants

This analysis uses survey data from 1180 women reporting on 3744 females in respondent households and 15,086 females in neighboring households across the four humanitarian settings named above. Multi-stage cluster sampling was used to select primary respondents for each of the four studies [2, 17, 18, 20]. A trained interviewer approached a selected house and asked to speak to the female head of the household. She explained the purpose of the interview, its anticipated duration, the assurance of anonymity and the need for privacy. If the woman identifying as female head of household gave her informed consent, the interview began in a private location chosen by the respondent. If the woman refused or was unable to speak to the interviewer privately, the interviewer thanked her for her time and moved to the next house identified by the sampling procedure.

### Study design

The Neighborhood Method is a population-based approach to measuring GBV that is based on a random sample of adult women reporting on their own experiences of GBV as well as the GBV experiences of others within their social networks [17]. This method was first adapted from the Sisterhood Method, a method for measuring maternal mortality using secondary reporting [21], and our study populations were further adapted in each of our study location sites based on learning from previous sites. Interviewers asked adult female respondents about their own GBV history (standard or self-report) and the experiences of their counterparts in the closest neighboring households (secondary report). In addition to asking about a neighboring adult female, interviewers in Liberia, Ethiopia, and Sri Lanka also asked about the experiences of all other women and children living within the respondents’ own household and the neighbor’s household (See Table 1). Technically, reporting of the prevalence of violence among children or women other than the respondent in the household is a form of secondary reporting, as the respondent is reporting on the experience of others. However, reporting about other members of a respondent’s household is a generally accepted survey methodology for largescale surveys like the Demographic Health Survey and Multiple Indicator Cluster Survey [22–24]. This study makes a distinction between this standard approach and the innovative approach of secondary reporting about the experience of other children and women living in a neighboring household. In Uganda, respondents were additionally asked to report on the experiences of their sisters.

**Table 1:** Summary of methodological features across the four studies

Countries	Context	Years of Data Collection	Populations of Interest for Household Survey/Standard Self-Reporting (n)	Populations of Interest for Neighborhood Method/Secondary Reporting (n)	Recall Period
Uganda	Internally displaced persons (IDP) camps	December 2006 to January 2007	Adult women (204)	Sisters (268)	12 months
				Adult female neighbors (1206)	
Liberia	Resettled communities in an urban county	June 2007 to August 2007	Adult women (600)	All females in respondents’ household (2460)	18 months
	Conflict-affected communities in a rural county			Adult female neighbor and all of the females in the neighbor’s household (10,287)	
Ethiopia/ Somalia	Refugee camps	June 2008 to July 2008	Adult women (244)	All females in respondents’ household (597)	18 months
	Host community			Adult female neighbor and all of the females in the neighbor’s household (2709)	
Sri Lanka	Villages	June 2008 to August 2008	Female age 16 or older (355)	All children in respondents’ household (845)	18 months
	Resettled villages IDP camps			Adult female neighbor andall of the children in the neighbor’s household (2364)	

After receiving informed consent from participants, trained local interviewers used a standardized protocol to ask respondents basic questions about their household demographics and those of their closest neighbors (as identified by the interviewer to eliminate potential bias). Interviewers then asked an open-ended question about the ‘biggest challenges facing women and girls in their community’. This question often resulted in respondents initiating a discussion on the topic of GBV and would be prompted later in the interview if not spontaneously raised. The study team drew upon the strengths of qualitative and quantitative methods by using a slow and semi-structured interview schedule to allow for enhanced trust-building between the interviewer and the respondent. The survey instrument measured three distinct forms of GBV: intimate-partner rape (defined as sexual intercourse, or attempted sexual intercourse, without consent by a husband or intimate partner), non-intimate partner rape (defined as sexual intercourse, or attempted sexual intercourse, without consent by someone other than a husband or intimate partner), and physical violence (defined as any act of non-sexual action that resulted in physical harm and was committed with the intent to do harm) [25]. Interviewers were trained to probe on incidents to ensure that they met the case definitions for GBV and used a bounded recall period from 1 year to 18 months. To reduce problems with telescoping, interviewers used important local or national landmark events to help respondents more accurately place the date of their experiences. Survey questions were designed to take on a conversational interview format interwoven with systematic questions about experiences of the population of interest to ensure consistency.

### Reliability and validity of secondary reporting

To examine the consistency of patterning and of overall rates of prevalence between primary and secondary reporting, we made three comparisons: incidence of violence self-reported by the respondent vs. (i) incidence reported by the respondent about her neighboring female head-of-household and (ii) incidence reported by the respondent about her sisters (Uganda study only). We also compared (iii) incidence of violence reported by the respondent about other women and children living in her own household vs. incidence reported by the respondent about other women and children in her neighbor’s household.

We assessed the reliability of secondary reporting across all study groups by calculating an intra-class correlation (ICC) coefficient. The coefficient was used to determine the amount of measurement error between secondary reporting using the Neighborhood Method and traditional self-reporting, and to gauge the extent to which secondary reporting could replace or reliably supplement traditional self-reporting. In this situation, we were interested in looking at overall *consistency of patterning*. To determine which variation of the ICC coefficient constituted the most appropriate measure, we used several pieces of information: for the comparison of self- and secondary reporting, a two-way analysis of variance for the prevalence of gender-based violence was deemed appropriate. The methods (i.e. secondary reporting and self-reporting) are considered “fixed” effects as they are the only methods of interest in this report [26]. The unit of analysis used were individual ratings. The two-way mixed, single measures intra-class correlation coefficient ICC [1, 3] is the best-suited coefficient for reliability analysis; an ICC between 0.9 and 1.0 is evidence for high reliability of the secondary reporting method compared to the self-report gold standard [26], and was used as our standard for measuring high reliability between secondary reporting and self-reporting.

In addition to examining the overall reliability of secondary reporting using the ICC, we also examined reliability at the level of individual interviews using additional data collected in the study of Somali refugees in Ethiopia. In that study, the research team conducted 23 ‘matched’ interviews in which two neighbors were asked to report on the experiences of violence for two common neighbors (Table 2). Cohen’s kappa was calculated to measure the agreement between the matched interviews beyond chance alone, thereby assessing the degree to which one respondent reporting on violence amongst her neighbors agreed with another respondent reporting on violence amongst the same neighbors.

**Table 2:** Cohen’s kappa statistics for 23 matched interviews about two common neighbors in Kebribeya Camp in Ethiopia

Type of GBV	Kappa statistic (95%CI)
Physical violence	0.45 (0.27, 0.63)
Rape	0.48 (0.46, 0.86)

We assessed the validity of the secondary reporting method by comparing results on incidences of violence from this new method with results from self-reporting and reporting about the respondents’ own household. Due to the underreported nature of GBV, it is difficult - even with traditional self-reporting methods - to confirm the extent to which a survey measure ascertains its true prevalence. Without a true ‘gold standard’, we utilized self-report of GBV as a proxy measure. Unlike above, where the analysis explored the *consistency of patterning*, this analysis assessed *correspondence in rates of prevalence* between primary and secondary reports. We performed a two-sample z-test to examine differences between proportions and assessed whether the reported prevalence of violence was different between primary and secondary reporting for each study sample and for each form of violence. If standard and secondary reported prevalence failed to show statistically significant differences at the 5% level, secondary reporting was considered to indicate sufficient correspondence in comparison to self-reporting or reporting about respondents’ own household.

## Results

### Reliability of secondary reporting

Table 1 presents the prevalence of self-reported and secondary-reported GBV (physical violence, intimate partner rape, and non-intimate partner rape). Using reported prevalence from all study groups, ICCs were calculated for each category of violence and for each of the three reporting population comparisons of interest (Table 3). ICCs for the comparison of respondent vs. neighbor head-of-household and for respondent household vs. neighbor household were generally high across all forms of violence, with all ICCs greater than 0.9, suggesting high reliability between secondary reporting and self-reporting [26]. This indicates that secondary reporting by neighbors is approximately as consistent as self-reporting in ascertaining experiences of GBV in households, and that it is consistent in its identification of households with higher or lower rates of violence. For the respondent vs. sisters comparison, however, reliability was poor, with all ICCs under 0.9, indicating that secondary reporting from sisters was less robust for reporting prevalence of GBV as self-reporting. This low ICC for sisters was likely affected by limited data, as comparison data for sisters was only collected at the Uganda sites. Additionally, reports for sisters may have been lower due to social or physical distance.

**Table 3:** Prevalence of self-reported and secondary reported gender-based violence and results of two-sample z-test for differences in prevalence by reporting population

Reporting population comparison	Type of violence	Study sample	Standard reported prevalence (n)	Secondary reported -prevalence(n)	z-test *p*-value
Respondent vs. neighbor head-of-household	Physical violence	Uganda	51..70% (201)	44.00% (1166)	**0..04**
	Physical violence	Liberia, Montserrado	76.70% (296)	69.20% (1170)	**0.01**
	Physical violence	Liberia, Nimba	83.30% (300)	75.70% (1178)	**0.01**
	Intimate partner rape	Uganda	40.50% (200)	25.10% (1109)	**< 0.01**
	Intimate partner rape	Liberia, Montserrado	76.70% (163)	66.90% (640)	**0.02**
	Intimate partner rape	Liberia, Nimba	81.60% (152)	72.00% (536)	**0.02**
	Non-intimate partner rape	Uganda	5.50% (201)	3.30% (1160)	0.12
	Non-intimate partner rape	Liberia, Montserrado	23.40% (295)	13.60% (1131)	**< 0.01**
	Non-intimate partner rape	Liberia, Nimba	33.70% (300)	24.20% (1135)	**0.01**
Respondent household vs. neighbor household	Physical violence	Liberia, Montserrado	45.00% (1264)	50.90% (4119)	**< 0.01**
	Physical violence	Liberia, Nimba	44.00% (1163)	50.90% (3640)	**< 0.01**
	Physical violence, women	Ethiopia, Aw Barre Town	57.60% (118)	57.90% (442)	0.96
	Physical violence, women	Ethiopia, Kebribeya Camp	63.60% (151)	49.40% (514)	**0.01**
	Physical violence, women	Sri Lanka, IDP sites	13.00% (177)	14.00% (486)	0.74
	Physical violence, women	Sri Lanka, villages	10.30% (252)	9.20% (721)	0.59
	Physical violence, boys	Sri Lanka, IDP sites	2.00% (152)	5.50% (433)	0.07
	Physical violence, boys	Sri Lanka, villages	6.70% (164)	3.60% (494)	0.10
	Physical violence, girls	Ethiopia, Aw Barre Town	29.20% (120)	28.50% (417)	0.89
	Physical violence, girls	Ethiopia, Kebribeya Camp	29.60% (152)	22.70% (446)	0.09
	Physical violence, girls	Sri Lanka, IDP sites	2.90% (139)	7.60% (344)	0.05
	Physical violence, girls	Sri Lanka, villages	3.40% (176)	3.70% (482)	0.84
	Intimate partner rape	Liberia, Montserrado	73.50% (268)	74.70% (958)	0.68
	Intimate partner rape	Liberia, Nimba	69.00% (197)	74.90% (582)	0.11
	Non-intimate partner rape	Liberia, Montserrado	19.70% (1240)	20.50% (3999)	0.53
	Non-intimate partner rape	Liberia, Nimba	23.30% (1145)	26.80% (3516)	**0.02**
	Non-intimate partner rape, women	Ethiopia, Aw Barre Town	43.00% (121)	42.00% (443)	0.85
	Non-intimate partner rape, women	Ethiopia, Kebribeya Camp	34.00% (150)	34.40% (514)	0.92
	Non-intimate partner rape, women	Sri Lanka, IDP sites	6.80% (176)	2.50% (476)	**0.01**
	Non-intimate partner rape, women	Sri Lanka, villages	3.20% (251)	3.30% (707)	0.96
	Non-intimate partner rape, girls	Ethiopia, Aw Barre Town	2.50% (120)	8.40% (417)	**0.03**
	Non-intimate partner rape, girls	Ethiopia, Kebribeya Camp	1.32% (152)	4.00% (446)	0.11
	Non-intimate partner rape, girls	Sri Lanka, IDP sites	0.00% (139)	0.30% (342)	0.52
	Non-intimate partner rape, girls	Sri Lanka, villages	0.00% (176)	0.20% (481)	0.55
Respondent vs. sister	Physical violence	Uganda	51.70% (201)	36.50% (266)	**0.01**
	Intimate partner rape	Uganda	40.50% (200)	22.10% (254)	**< 0.01**
	Non-intimate partner rape	Uganda	5.50% (201)	3.40% (263)	0.28

Finally, when ICCs were calculated in sub-group analyses for adult women ≥18 years of age and girls < 18 years of age, a lower ICC of 0.722 was found for reports of rape perpetrated against girls in a respondent’s household vs. a neighboring household as shown in Tables 4 and 5. To assess reliability at the level of individual interviews, 23 matched interviews were performed in Kebribeya Camp in Ethiopia, wherein two neighbors were asked to report on a third, common neighbor. Forty-two out of 74 reports of physical violence within the recall period (57%) ‘matched,’ or were simultaneously reported by two neighbors about the same third neighbor. A total of 35 incidents of rape were reported among the matched interviews. Fifteen of these 35 incidents of rape (43%) were reported by both neighbors about the same third neighbor. Cohen’s kappa statistics for both physical violence and rape (Table 6) show statistically significant (*p* < 0.001) ‘moderate’ agreement between matched neighbor reporting, suggesting, in this case, moderate reliability or *consistency in patterning* between both secondary reporting and self-reporting [27].

**Table 4:** Intra-class correlation coefficients for comparisons of three different types of secondary reporting vs. standard reporting among women (≥ 18 years of age)

Reporting population comparison	Type of violence	ICC
Respondent household vs. neighbor household	Physical violence	0.97
	Intimate partner rape	–
	Non-intimate partner rape	0.99

“---” denotes no self-reported concordant data available for women and girls for intimate partner rape in sub-group analyses between respondent households versus neighbor households

**Table 5:** Intra-class correlation coefficients for comparisons of three different types of secondary reporting vs. standard reporting among girls (< 18 years of age)

Reporting population comparison	Type of violence	ICC
Respondent household vs. neighbor household	Physical violence	0.97
	Intimate partner rape	–
	Non-intimate partner rape	0.72

“---” denotes no self-reported concordant data available for women and girls for intimate partner rape in sub-group analyses between respondent households versus neighbor households

**Table 6:** Intra-class correlation coefficients for comparisons of three different types of secondary reporting vs. standard reporting

Reporting population comparison	Type of violence	ICC
Respondent vs. neighbor head-of-household	Physical violence	0.98
	Intimate partner rape	0.98
	Non-intimate partner rape	0.94
Respondent household vs. neighbor household	Physical violence	0.99
	Intimate partner rape	0.99
	Non-intimate partner rape	0.99
Respondent vs. sister	Physical violence	0.22
	Intimate partner rape	0.87
	Non-intimate partner rape	0.29

### Validity of secondary reporting

Table 1 presents the results of two-sample z-tests for proportions to assess overall correspondence in rates of prevalence i.e. whether reported prevalence of violence was significantly different between reporting populations. The results of this analysis were mixed. For secondary reporting about neighbor head-of-household compared to self-report, all but one study sample were found to have significantly lower secondary-reported rates of violence. This finding was observed for all three forms of GBV across three different study settings. Non-intimate partner rape assessed in Uganda showed no significant difference in the prevalence reported about neighbors compared to the self-reported prevalence.

For secondary reporting about neighboring households compared to respondents’ households, there were no significant differences for 18 out of 24 such comparisons, suggesting more consistent *correspondence in rates of prevalence*.

Of the six comparisons of respondents’ households vs. neighbors’ households that did show statistically significant differences in GBV, two study samples (involving physical violence against adult women in Ethiopia and non-intimate partner rape against adult women in Sri Lanka) had lower prevalence of violence reported in the neighbors’ households. For the remaining four study samples (two of physical violence against women and girls in Liberia, one of non-intimate partner rape in Liberia, and one of rape of girls in Ethiopia), higher prevalence of violence was reported in the neighbors’ households compared to the respondents’ households.

Finally, secondary reporting about respondents’ sisters (data collected in Uganda only) yielded statistically significantly lower prevalence rates than self-reporting for physical violence and rape by an intimate partner. No statistically significant difference was found for the less frequent reporting of non-intimate partner rape.

## Discussion

With the lack of any clear basis for establishing a ‘gold standard’ of prevalence of sexual violence and clear potential for risks associated with reporting in insecure environments, surveys of violence against women in humanitarian settings are widely seen as likely to involve under-reporting. This limitation has also been documented in wealthier countries [12], and under-reporting was indicated in our one study where we asked different women about violent events in the same neighboring household. This suggests that the frontier of advancing the science of documenting GBV may not involve having a perfect survey method that captures all events. Instead, the objective perhaps should be to have complete enough documentation to understand the magnitude and various kinds of violence occurring in a specific setting and to record it with a reproducible process that will document changes over time. This approach of monitoring that misses some cases but captures patterns and eruptions has served the polio and smallpox eradication programs well [28, 29]. Similar patterning was also noted with the original use of the Sisterhood Method [30, 31], and a recent attempt to use lot quality assurance sampling (LQAS) to measure GBV in emergencies [32]. To this end, the Neighborhood Method seems to perform well compared with the standard household survey.

The results presented above illustrate that ICCs showed a generally high level of consistency in identifying individuals and households at higher *and* lower risk for GBV. However, in terms of estimated prevalence of GBV based on primary and secondary reports, there is clear variation with respect to the reporting population that is being addressed. Amongst women reporting on themselves and their neighbors, secondary reporting on neighbors generally resulted in a lower estimate of prevalence than self-report. In contrast, prevalence estimates based upon secondary reports of GBV in neighbors’ households and reports of GBV within the respondents’ household showed much higher levels of statistical correspondence.

The lower prevalence estimates for neighbors vs. selves may represent a respondent’s lack of knowledge about her neighbor’s experience with GBV or a bias against disclosing information about one’s neighbors. Although it is theoretically possible that the higher incidence from self-reporting could reflect that the standard self-reporting approach may be biased towards over-reporting, this is unlikely given the literature showing that GBV tends to be underreported due to stigma and other negative repercussions for the survivor [3, 7, 8].

For the comparisons between a respondent’s household and prevalence reported about the neighbor’s household, we note that the standard approach is in fact a form of secondary reporting, as respondents are asked to report on other women and children living in their own household. Data about the respondent’s household is similarly based on the assumption that an adult female has complete and accurate information about the women and children living under her care. This information is, therefore, also subject to similar biases of knowledge, non-disclosure, and social desirability as the respondent likely views herself as being responsible for the wellbeing and safety of others in the same household. Reporting about one’s household, however, still reflects a common and standard approach for assessing population health in conflict and development settings, and we thus compare it with the novel approach of asking about the respondents’ neighbor’s household. For comparisons between ‘standard’ secondary reporting about respondents’ households vs. novel secondary reporting about neighboring households, statistical tests for the most part failed to detect any significant difference in the prevalence of GBV, suggesting correspondence in overall rates of prevalence and that secondary reporting about neighboring households may be as valid as reporting about respondents’ own households on GBV.

Additionally, in cases where there was less consistency in patterning between secondary reporting and self-reporting, higher reported rates of violence in neighboring homes for children suggest that this novel type of secondary reporting may foster higher rates of disclosure, especially if social desirability bias prevents females from reporting events in their own households. These findings raise the potential that secondary reports on neighbor’s children may be more reflective of the truth than self-report on children in the respondent’s household. If supported by additional testing, this finding could have important implications for measuring violence against children – a growing measurement trend – and suggests that secondary reporting has the potential to reveal better data for younger populations than current assessments.

Taking both reliability and validity into consideration, overall findings suggest similar levels of reporting between the Neighborhood Method and standard self-reporting *when looking across household data*. The possibility exists that the consistency between self-reported household rates vs. neighbor rates involves under-reporting on both populations through true limitations of knowledge or reluctance on the part of the interviewee. There was little data available for us to assess the use of secondary reporting on respondents’ sisters’ experiences, as we did not use this form of reporting outside of Uganda. One barrier to collecting this data in other settings is the low likelihood of sisters knowing about each other’s experiences, especially for sensitive topics such as GBV, where chronic civil unrest and large population movements may limit communication and such intimate knowledge. Other factors that may influence the variability in patterns of difference between standard and secondary reported GBV incidence include the geographic distribution of households, where rural households in some settings may be too dispersed for respondents to accurately know their neighbors’ experiences, and cultural norms in different populations that are relevant to disclosure of GBV.

Limitations of this analysis include the lack of a true ‘gold standard’ for validity testing, such that self-reporting methods as means of measuring GBV incidence is not itself definite. We are thus limited to conducting validity testing for the non-inferiority of secondary reporting compared to the usual, standard approach. While our primary and secondary samples were powered to compare prevalence rates, we acknowledge that other factors such as internal variation and larger confidence intervals will also factor into whether our comparisons showed significance. Finally, the four studies included in this paper all focused on gender-based violence, thus limiting the generalizability of the results to understand the use of secondary reporting for other population health concerns.

## Conclusion

In a humanitarian culture driven by an imperative to deliver assistance, often at the expense of rigorous assessment and evaluation, alternative measurement approaches better suited to contexts of war and disaster are needed. Without some rate-based measure of GBV, trends or assessments of preventative measures will not be easily evaluated. This analysis offers important initial insights into the reliability and validity of secondary reporting as a tool for field epidemiologists in humanitarian settings. Further exploration of secondary reporting will strengthen our understanding of whether and when secondary reporting is a viable alternative or supplement to standard methods.

## Data Availability

The datasets used and/or analyzed during the current study are available from the corresponding author upon reasonable request.

## Abbreviations

GBV: Gender-based violence
ICC: Intra-class correlation
IPV: Intimate partner violence
